# Hyperoxia post cardiac arrest: experience of a UK ICU

**DOI:** 10.1186/cc9724

**Published:** 2011-03-11

**Authors:** L Tameem, K Rooney, S Deep, M Thomas

**Affiliations:** 1 Bristol Royal Infirmary, Bristol, UK

## Introduction

A recent US multicentre study demonstrated an increased mortality in intensive care patients exposed to high arterial oxygen levels following return of spontaneous circulation (ROSC) after cardiac arrest [1]. We attempted to ascertain the incidence of hyperoxia and associated mortality in a similar cohort of patients in the UK.

## Methods

We performed a retrospective observational study of a computerised database (Draeger Innovian) over a 14-month period (March 2009 to May 2010). All adult, nontraumatic cardiac arrests within 24 hours of admission to the ITU were included. Sixty-nine patients were identified. The following data points were analysed: FiO_2_, pO_2 _and outcome. Time to first ABG and the PaO_2_/FiO_2 _(P/F) ratio were calculated. As per the US study, hypoxia was defined as a pO_2 _< 60 mmHg or P/F ratio <300; hyperoxia as PaO_2 _> 300 mmHg. Normoxia was the values in between.

## Results

Ninety per cent of patients had an arterial blood sample within the first hour after admission, compared with the US study where 27.5% of patients did not receive an arterial sample within the first 24 hours. Hyperoxia was only half as common in our population and was associated with the lowest mortality rate (50%). This is at odds with the Kilgannon study, which showed that hyperoxia was associated with the highest mortality [1]. Using their definition of hypoxia, there is no significant difference in mortality between hypoxia and normoxia in our study. If hypoxia is defined as pO_2 _< 60 mmHg then the hypoxia rate is only 2.9% with a mortality rate of 100%.

## Conclusions

In a single UK adult ICU attached to a cardiac arrest centre, hyperoxia after cardiac arrest was uncommon and associated with the lowest mortality. This is associated with increased vigilance in measuring arterial blood gases. Recent guidelines from the Resuscitation Council advise that inspired oxygen should be titrated to achieve a SaO_2 _of 94 to 98% due to potential harm from hyperoxia [2]. This assertion is not borne out by our data. The definition of hypoxia is important as there is a significant difference in both incidence of hypoxia and mortality rates dependent on whether the P/F ratio is considered. In practical terms, clinicians can only aim to optimise their arterial oxygen saturations, not the P/F ratios.
